# Sex, horizontal transmission, and multiple hosts prevent local adaptation of *Crithidia bombi*, a parasite of bumblebees (*Bombus* spp.)

**DOI:** 10.1002/ece3.250

**Published:** 2012-05

**Authors:** Silvio Erler, Mario Popp, Stephan Wolf, H Michael G Lattorff

**Affiliations:** 1 Institut für Biologie, Molekulare Ökologie, Martin-Luther-Universität Halle-WittenbergHoher Weg 4, 06099 Halle (Saale), Germany; 2 Present address: Departamentul de Tehnologii Apicole şi Sericicole, Universitatea de Ştiinţe Agricole şi Medicină VeterinarăCalea Mănăştur 3–5, 400372, Cluj-Napoca, Romania; 3 Present address: Department of Plant and Invertebrate Ecology, Rothamsted ResearchAL5 2JQ Harpenden, United Kingdom

**Keywords:** *Bombus*, bumblebee, coevolution, *Crithidia bombi*, host-parasite interaction, population genetic structure

## Abstract

Local adaptation within host-parasite systems can evolve by several non-exclusive drivers (e.g., host species-genetic adaptation; ecological conditions-ecological adaptation, and time-temporal adaptation). Social insects, especially bumblebees, with an annual colony life history not only provide an ideal system to test parasite transmission within and between different host colonies, but also parasite adaptation to specific host species and environments. Here, we study local adaptation in a multiple-host parasite characterized by high levels of horizontal transmission. *Crithidia bombi* occurs as a gut parasite in several bumblebee species. Parasites were sampled from five different host species in two subsequent years. Population genetic tools were used to test for the several types of adaptation. Although we found no evidence for local adaptation of the parasite toward host species, there was a slight temporal differentiation of the parasite populations, which might have resulted from severe bottlenecks during queen hibernation. Parasite populations were in Hardy-Weinberg equilibrium and showed no signs of linkage disequilibrium suggesting that sexual reproduction is an alternative strategy in this otherwise clonal parasite. Moreover, high levels of multiple infections were found, which might facilitate sexual genetic exchange. The detection of identical clones in different host species suggested that horizontal transmission occurs between host species and underpins the lack of host-specific adaptation.

## Introduction

Coevolution might occur either in beneficiary relationships as mutualisms or symbioses, or in antagonistic relationships as host-parasite systems. Initially, a first step toward a coevolutionary pattern occurs via local adaptation. Genotypes might adapt to the local abiotic or the biotic environmental conditions. Given opposing interests of parasites and their hosts selection pressure, and thus adaptation, can be assumed to be particularly pronounced in host-parasite systems.

Transmission and virulence of parasites are crucial factors shaping any type of host-parasite adaptation. The effects of both factors on the evolution of host-parasite adaptation become increasingly complex with more sophisticated life cycles. In social host species, especially eusocial insects, colony structure, overlapping generations, and colony life cycles may strongly affect parasite adaptation (Schmid-Hempel 1998). Social insect colonies typically provide stable abiotic and biotic conditions in form of homoeostasis, high genetic viscosity (genetic makeup of the colony determined by the genotype of the queen), large amounts of brood and food resources, which make them prone to attacks by parasites and pathogens (Schmid-Hempel 1995). Additionally, the high genetic viscosity of colonies as compared to non-social hosts provides a high density of genetically relatively similar individuals within a colony available for a considerable time. This may not only lower the challenges for the parasite to propagate successfully once being transmitted to a new host individual but also increases the rate of transmission of parasites between individuals (Schmid-Hempel 1998).

Transmission of pathogens in populations of social insect colonies occurs at three levels: (1) from the sexuals to the offspring (vertical); (2) among individuals, of the same and/or different sex or caste (horizontal/vertical); and (3) between colonies (horizontal). Here, especially the second point (among castes) may play an important role, as it is nearly exclusively found in social insects (Schmid-Hempel 1998).

The bumblebees *Bombus* spp. and the obligatory gut parasite *Crithidia bombi* (Lipa and Triggiani 1988) are a model system for the study of host-parasite interactions (Schmid-Hempel 1998). Bumblebees are primitively eu-social insects with an annual colony life cycle, with only young mated queens entering hibernation and founding the next generation of colonies in spring (Sladen 1912; Goulson 2010). After the first batch of workers has emerged, the queen exclusively devotes herself to reproduction whereas workers maintain the nest, forage for food, and care for the brood (Sladen 1912; Goulson 2010). At the end of the season, males and females are produced. After mating, queens enter hibernation. As queens typically mate only once (Estoup et al. 1995; Schmid-Hempel and Schmid-Hempel 2000), intracolonial relatedness among female offspring (i.e., workers and newly raised queens) is high at 75%, potentially facilitating parasite establishment within a colony, if the genotype of the host is important for the susceptibility to a parasite.

*Crithidia bombi* is a widespread, chronic gut pathogen of bumblebees that nevertheless might reduce the fitness of bumblebee queens drastically (Brown et al. 2003). The pathogen affects ovarian development as well as early colony development (Shykoff and Schmid-Hempel 1991a), but hibernating queens are not directly affected by *C. bombi* (Shykoff and Schmid-Hempel 1991b; Yourth et al. 2008).

The main infection pathway of bumblebees by *C. bombi* is by ingestion of infectious cells in the colony (vertical transmission). This is strongly influenced by the number of individuals available as potential vectors, hence by the colony size. Within the host, *C. bombi* typically reproduces clonally, but recent experimental studies showed that the parasite also reproduces sexually (Schmid-Hempel et al. 2011). Foraging workers may additionally acquire infections on flowers via horizontal transmission of *C. bombi* (Durrer and Schmid-Hempel 1994). This colony-to-colony dispersal of the parasite depends on both numbers of foragers per colony (i.e., colony size) and forager frequency on commonly exploited flowers and may even allow transmission across bumblebee species. However, bumblebee species with different tongue length typically have a small niche overlap (Goulson and Darvill 2004) and horizontal infections across different species can be assumed to correlate with niche overlap.

Though these factors shape the probability that a bumblebee encounters a parasite, they only insufficiently reflect the rate of successful infections. Foraging bumblebees may be able to detect and avoid contaminated flowers, especially when encountering coadapted pathogens (Fouks and Lattorff 2011).

Once being ingested, the host (colony) genotype may play a crucial role for the parasite establishment. At the population level, the prevalence of *C. bombi* has been shown to be negatively associated with overall host heterozygosity (Whitehorn et al. 2010). Bumblebee populations exhibiting low genetic diversity, possibly due to inbreeding, show a higher prevalence of *C. bombi* than populations having high levels of heterozygosity (Whitehorn et al. 2010). More specifically, the host genotype may affect the ability to respond to *C. bombi* infection by activating their innate immune system and up-regulate effector genes (e.g., antimicrobial peptides [AMPs], lysozymes, serine proteases) (Riddell et al. 2011). AMPs are up-regulated within hours after parasite or pathogen exposure (Erler et al. 2011), but can be highly specific in their action with significant genotype by genotype interactions for the expression profile (Riddell et al. 2009).

However, a high prevalence of *C. bombi* of 80% of the individuals per colony during the middle of the season (Shykoff and Schmid-Hempel 1991b; Gillespie 2010; Popp et al. 2012) suggests an overall efficient spread of the parasite within the colony (vertical) in combination with high horizontal transmission. In contrast to the high degree of infection in workers, only 5–10% of the young queens are infected (Ulrich et al. 2011) and if so, infections with multiple *Crithidia* strains are rare. This has been explained by a process of strain filtering in workers (Ulrich et al. 2011) possibly accumulating strains due to subsequent infections. Both the low number of infected hibernating queens and the low frequency of multiple infections among these queens constitute a potentially severe bottleneck for *C. bombi* populations.

Although *C. bombi* can infect many bumblebee species, little is known about genotypic interactions between multiple hosts and multiple parasite infections. Under natural conditions, parasites may be adapted to their hosts in broad or narrow ranges (Lajeunesse and Forbes 2002). Local adaptation between *Bombus terrestris* and *C. bombi* during one infection cycle has not been detected (Imhoof and Schmid-Hempel 1998). However, using mixed *C. bombi* strains from different colonies for serial infections of different bumblebee colonies, local *C. bombi* strain infections result in decreased cell numbers in bumblebees of the parasite alien colony (Yourth and Schmid-Hempel 2006). Thus, local adaptation was observed under experimental conditions for related bumblebee colonies, which can be explained by genotype-genotype interaction between host and parasite.

Here, we test for the significance of genetic effects (host species) and temporal effects (years) on local host-parasite adaptations by screening five different bumblebee species, in two subsequent years. This allowed us for assessing any local host-parasite adaptations and also *C. bombi* prevalence with respect to ecological, genetic, and demographic factors.

## Material and Methods

### Bumblebee samples

Workers and males (drones) from five different bumblebee species, *B. terrestris*, *B. hortorum*, *B. pascuorum*, *B. lapidarius*, and the cuckoo bumblebee *B.* (*Psithyrus) vestalis*, were collected during foraging on flowers in Halle (Saale), Germany, in two similar sites, which served as replicates; the botanical garden (51°29′21.52″N; 11°57′37.36″E) and an urban flower rich park (51°29′27.55″N; 11°56′11.54″E) in 2008 and 2009 (overview see Table S1). Samples for both years were taken in the first week of July. Both sampling sites are separated from each other by 2 km, thus well within the flight range of workers, males, and queens (*B. terrestris*: Kraus et al. 2009; *B. terrestris*/*B. lapidarius*: Wolf et al. 2012). Bumblebee species were identified using the taxonomic key of Mauss (1994).

### Bumblebee genotyping

Genomic DNA was extracted from a hind leg per individual using a modified Chelex protocol (Erler and Lattorff 2010). The genotyping multiplex PCR primer set (fluorescent-labeled B10, B11, B124, B126, and B100) is based on Estoup et al. (1995, 1996). PCR amplification and analysis of fragment sizes followed the protocol from Erler and Lattorff (2010) using a MegaBACE 1000 Sequencer (GE Healthcare, Munich, Germany) for DNA fragment separation and the MegaBACE Fragment Profiler software for genotyping.

### DNA extraction of *C. bombi*

Nuclear DNA of the parasite *C. bombi* was extracted using a modified Chelex protocol (Walsh et al. 1991) by homogenizing the abdomen (samples of 2008) or the gut (samples of 2009) of each individual in 500 and 300 μL aqua dest, respectively. A total of 200 μL of each homogenate was centrifuged at 3220 *g* for 30 min. The supernatant was discarded and the remaining pellet was homogenized in 100 μL 5%-Chelex solution (Bio-Rad, Munich, Germany) and 5 μL 1% proteinase K was added. Samples were processed in a thermocycler using the following programe: 1 h at 55°C; 15 min at 99°C; 1 min at 37°C, and a final step for 15 min at 99°C. DNA was stored at –20°C until further processing.

### *Crithidia bombi* genotyping

Fluorescence-labeled primers (Cri 4, Cri 1.B6, Cri 2.F10, and Cri 4G9) were used in a single multiplex PCR as in Schmid-Hempel and Reber Funk (2004) (4G9, R. Schmid-Hempel, pers. comm.). A PCR reaction (10 μL) comprised 1 μL template DNA, 5 μL PCR Master Mix (Promega, Madison, WI), 2.2 μL aqua dest, 0.3 µM of each forward and reverse primer for Cri 4 and Cri 2.F10, and 0.15 µM of each forward and reverse primer for Cri 1.B6 and Cri 4G9. The PCR reactions were run in a PE 9700 thermocycler (Perkin Elmer, Waltham, MA, USA) with the following program: 4 min denaturation at 95°C, then 35 cycles with 1 min, 95°C; 30 sec, 53°C; 30 sec, 72°C, and final elongation at 72°C for 4 min. Allele sizes were analyzed with MegaBACE 1000 Sequencer (GE Healthcare) and assigned using the software MegaBACE Fragment Profiler.

### Data analysis and statistics

#### Bumblebee sibship reconstruction

The software COLONY version 1.3 (Wang 2004) was used for sibship reconstruction of bumblebee samples. Based on a maximum likelihood approach, this software uses both the individual genotypes (haploid and diploid) and the overall allele frequencies in the sample to infer the minimum number of putative natal colonies. In order to correct for the number of non-detected colonies due to finite sample sizes, the non-sampling error (NSE) was estimated by fitting the observed distribution to a truncated Poisson distribution (Kraus et al. 2009; Goulson et al. 2010).

Analyzed microsatellite parameters (e.g., allelic richness, observed and expected heterozygosity, fragment size ranges) for the five different host species were either based on the genotypes of collected bumblebees or on the inferred queen genotypes. Expected and observed heterozygosity was determined with GENEPOP version 4.0 (Raymond and Rousset 1995; Rousset 2008), allelic richness by using HP-RARE 1.0 (Kalinowski 2005).

A generalized linear model (binomial distribution, logit-link function) implemented in STATISTICA 8.0 (StatSoft, Tulsa, OK) was used to estimate the impact of host species and sampling year on the prevalence of *C. bombi*. The same method was applied to test for a significant difference between the two sites.

#### Host-parasite coevolution

A reconstruction of the genealogical relationships was used to analyze congruence between the host and the parasite genealogies. The reconstructed queen genotypes of the five different bumblebee host species were used to construct distance matrices using the microsatellite distance program Microsat.c version 1.5e (http://hpgl.stanford.edu/projects/microsat/). Similarly, the distance matrices for the parasites were constructed using an estimate of the allele frequencies of all *C. bombi* genotypes extracted from a certain host species. Distance matrices were calculated based on number of shared alleles. –ln (proportion of shared alleles) transformed distances were used in the Clustering Calculator software (http://www2.biology.ualberta.ca/jbrzusto/cluster.php) with the Saitou and Nei Neighbour Joining algorithm to reconstruct a genealogical tree. Congruence between the two trees was tested and visualized using TreeMap version 1.0a (R. D. M. Page 1995, http://taxonomy.zoology.gla.ac.uk/rod/treemap.html) using the default costs for evolutionary events (codivergence = 0, host switching = 1, duplication = 1, and loss = 1).

As the previous analysis pools all host and parasite genotypes over years and sites additional data analysis was processed focusing on a finer scale to determine which factors influence parasite genotypic variance in host populations. As the allele frequencies in multiple infected hosts could not be unambiguously translated into individual genotypes, all further analyses were based on *C. bombi* genotypes from single *Crithidia*-infected individuals. Pairwise *F*_ST_ values were calculated for host species, sampling sites, and sampling year, using GENEPOP version 4.0 (Raymond and Rousset 1995; Rousset 2008). Parasite populations extracted from the respective host populations were analyzed similarly. Correlation analysis of corresponding *F*_ST_ values from host and parasite populations was done using STATISTICA 8.0.

In order to assess the population genetic structure of *C. bombi* populations, two different approaches were applied. The Bayesian clustering algorithm STRUCTURE (Pritchard et al. 2000) was used to reveal any hidden population structure, which might occur due to host genotypes or sampling year. We performed 10 replicate-runs with STRUCTURE varying the prior number of population to be expected ranging from *k*= 1 to *k*= 10. The number of populations represented by the *C. bombi* genotypes was analyzed from visual inspections of the graphic representation of the results as well as using the likelihood estimator for the varying number of populations.

*Crithidia bombi* genotypes from different host populations were used for an AMOVA (Arlequin version 3.0; Excoffier et al. 2005) in order to assess any host species specific structure in the distribution of genotypes. This type of analysis was also used to identify any temporal pattern in the distribution of *C. bombi* genotypes. Testing for significant differences, as shown by Salathé and Schmid-Hempel (2011), between the two sites was also performed by using AMOVA. Finally, we tested three different scenarios: (1) separated into host species neglecting spatial and temporal effects, (2) temporal separation neglecting species and spatial effects, and (3) spatial separation neglecting host species and temporal effects. As the experimental design is not hierarchical and not full-factorial, a single analysis focusing on all effects was not possible. Bonferroni correction was used to correct of multiple tests adjusting the significance level to *P*= 0.017.

#### Determination of clonal genotypes and horizontal transmission

As only *C. bombi* genotypes from bumblebees infected with a single strain of *Crithidia* were taken into account, genotypes sampled several times from different host individuals were determined. To assess asexual propagation of *C. bombi*, we used MLGsim (Stenberg et al. 2003) to determine the probability of a genotype sampled more than once to be clonal and not produced by sexual reproduction. Based on the sample size, the frequency of microsatellite alleles of the target genotype and the population-wide allele frequencies MLGsim simulates populations and calculates probabilities for all clonal genotypes found during simulations, in order to determine a significance level. The distribution of the probabilities was used to calculate a critical probability, with values below this level significant for a given nominal *P*-level (10^6^ simulations were used with a nominal significance level of *P*= 0.05). For samples with missing data (locus Cri4 or locus Cri4G9), the probability of the genotype was calculated from the remaining loci and simulations were run separately to determine the critical probability and the significance level. Finally, the genotypes occurring more than once were analyzed with respect to the host species from which they were extracted in order to determine the level of horizontal transfer between host species.

## Results

### Test for host-parasite local adaptation

**Figure 1:**
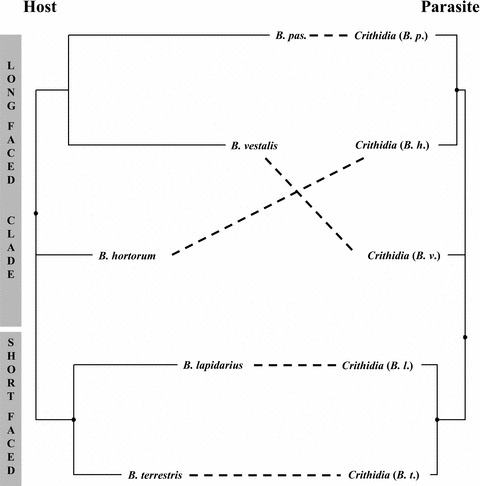
Genealogical analysis of potential genotype-genotype associations between different *Crithidia bombi* populations and its different host populations (*B. pas*., *Bombus pascuorum*).

Highly polymorphic microsatellite markers for the host (five; Table S2) and the parasite (four) populations allowed for the reconstruction of genealogical trees based on the allele frequency estimates. Pooled samples of bumblebees per species, years, and sites and pooled samples of *C. bombi* populations (single and mixed infections) showed a similar topology for both trees (Fig. 1). The host species tree matched the expected phylogenetic clusters for the five different bumblebee species, separated in short-faced (*B. lapidarius*, *B. terrestris*) and long-faced bumblebees (*B. hortorum*, *B. pascuorum*, *B. vestalis*) (Cameron et al. 2007).

The parasite tree is characterized by short branch lengths between the five different *C. bombi* populations (Fig. 1). The *C. bombi* population from *B. vestalis* was placed between the *C. bombi* cluster of *B. hortorum*/*B. pascuorum* and the cluster *B. lapidarius*/*B. terrestris*.

Three methods were used to characterize population differences and to test for significant differences among the five different *C. bombi* populations, because the similar topology of the host-parasite tree might indicate coevolution: (1) Pairwise *F*_ST_ for host and parasite populations; (2) Bayesian clustering analysis; and (3) analysis of genetic differentiation.

1. Genetic distances calculated on single *Crithidia*-infections showed no sign for local adaptation. Pairwise *F*_ST_ values between host and parasite populations did not correlate (*r*=–0.019, *N*= 91, *P*= 0.852). Maximum *F*_ST_ values of 0.607 were found between host species, whereas between parasites populations *F*_ST_ a maximum of 0.189 was detected, but most values being close to zero (Table S3).
2. Using the Bayesian clustering algorithm of STRUCTURE, only slight evidence for a differentiation of the populations was found, with respect to the sampling years. Multiple runs of STRUCTURE varying the prior number of populations resulted in *k*= 2 populations (log-likelihood =–1064.4). However, log-likelihood values for *k*= 1 to *k*= 3 populations are very close to each other and differentiation was not possible.
3. Finally, the genetic differentiation of *C. bombi* populations isolated from different host species was assessed using an AMOVA. Significant effects of differentiation were assessed for samples taken in two different years (*P*= 0.004). There were no significant effects with respect to host species or sites (*P* > 0.05). Results are combined in Table 1.

**Table 1:** AMOVA results for the effect of host species, year, and site. *P*-values have been adjusted (*P*= 0.017) due to multiple testing by 1023 permutations. Significant values are in bold.

Source	df	SSD	*P*-value
AMOVA for effect of host species			
Among host species	4	2.412	0.874 ± 0.010
Among populations within host species	9	7.653	0.076 ± 0.007
Among individuals within populations	89	58.557	0.953 ± 0.007
Within individuals	103	76.500	0.932 ± 0.008
Total	205	145.121	
AMOVA for effect of site			
Among sites	1	0.866	0.409 ± 0.018
Among populations within sites	12	9.198	0.142 ± 0.009
Among individuals within populations	89	58.557	0.954 ± 0.007
Within individuals	103	76.500	0.907 ± 0.010
Total	205	145.121	
AMOVA for effect of year			
Among years	1	2.582	**0.004 ± 0.002**
Among populations within years	12	7.482	0.572 ± 0.014
Among individuals within populations	89	58.557	0.948 ± 0.007
Within individuals	103	76.500	0.929 ± 0.008
Total	205	145.121	

Summarizing these results, no significant correlation for host and parasite *F*_ST_, no sign for population differentiation and no significant effects for species indicate that local adaptation may not occur for the five different bumblebee species and their respective trypanosome parasites.

### *Crithidia bombi* reproduction and genotype dispersal

If the main form of reproduction in *C. bombi* were clonal, the parasite populations should be highly structured with clear deviations from Hardy-Weinberg equilibrium (HWE) and strong linkage disequilibria (LD). However, a global analysis of all available *C. bombi* genotypes extracted from the single *Crithidia*-infected individuals showed no significant deviations from HWE with *P*-values ranging from 0.065 to 0.518 (2008) and 0.243 to 0.998 (2009) (Table 2A). Likewise, none of the pairwise comparisons of the four loci showed significant LD with *P*-values ranging from 0.145 to 0.998 (2008) and 0.077 to 0.661 (2009) (Table 2B).

**Table 2:** Results of the statistical analysis for deviation from Hardy–Weinberg equilibrium (A) and linkage disequilibrium (B), for the four different polymorphic loci of the trypanosome *Crithidia bombi*. Values are *P*-values. Only parasite genotypes from single *Crithidia*-infected bumblebees were included.

A		
Locus	2008	2009
Cri 1.B6	0.256	0.998
Cri 2.F10	0.518	0.911
Cri 4	0.152	0.243
Cri 4G9	0.065	0.376

**B**		
Locus versus locus	2008	2009

Cri 1.B6/Cri 2.F10	0.742	0.653
Cri 1.B6/Cri 4	0.998	0.077
Cri 2.F10/Cri 4	0.976	0.661
Cri 1.B6/Cri 4G9	0.145	0.246
Cri 2.F10/Cri 4G9	0.430	0.261
Cri 4/Cri 4G9	0.972	0.099

**Figure 2:**
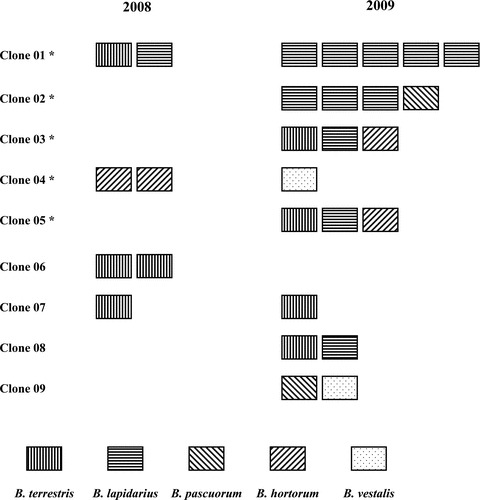
Association of multiple occurring clones within the five different host species and the two sampling years (2008, 2009). Each box represents one individual of the respective host species from which a parasite genotype has been extracted. Values for each single clone indicate the likelihood that the genotype represents a true clone, based on the allele frequencies of the multi-locus genotype: Clone 01: 4.8 × 10^−15^; Clone 02: 8.6 × 10^−8^ (locus Cri4G9 missing); Clone 03: 1.7 × 10^−12^; Clone 04: 6.4 × 10^−10^; Clone 05: 3.8 × 10^−7^; Clone 06: 9.4 × 10^−4^ (locus Cri4 missing); Clone 07: 3.8 × 10^−5^; Clone 08: 2.3 × 10^−2^ (locus Cri4G9 missing); and Clone 09: 3.5 × 10^−2^ (locus Cri4G9 missing). *Significant values for the likelihood that a genotype represents a clonal type.

A comparison of *C. bombi* genotypes showed a high diversity with 84 distinct genotypes to infer. Of those, 75 were singletons and nine occurred more than once. Two different approaches were used to estimate the number of non-sampled genotypes, since the observed number of genotypes which is restricted due to the finite sample size, might not represent the true number of genotypes circulating in the tested of bumblebee populations. Using the approach developed by Cornuet and Aries (1980), which assumes a binomial sampling distribution, a total number of 242 (NSE = 158) genotypes were estimated, whereas a fitted truncated Poisson distribution revealed 119 (NSE = 35) genotypes to be present. Multiple occurring *C. bombi* genotypes appeared in two to seven cases and were rarely restricted to a single species (two out of nine) (Fig. 2). The seven remaining genotypes occurred in two or even three different species. Testing for the clonal nature of these genotypes (see Fig. 2), we found at least five of the nine clonal genotypes are real clones. Based on the allele frequency of those clones, a low probability was calculated, that excludes their co-occurrence by chance. Non-significant genotypes had either low sample size (two) or low number of microsatellite loci contributing to the multi-locus genotype (three instead of four) (Fig. 2). One genotype was exclusively found in 2008, five were exclusively found in 2009, and three genotypes were present in both years.

### Horizontal versus vertical transmission: the impact of the environment

The analysis of matching topologies of the genealogical trees indicated a weak association of *C. bombi* genotypes and host species. Obviously, the host tree, based on genotypic markers, showed a separation of species with respect to ecologically important traits, such as tongue length. The prevalence of this parasite in different host species indicates a link between ecologically relevant factors that might influence the transmission of the parasite. A generalized linear model (GLZ) showed that host species (*P* < 0.001) and sampling year (*P* < 0.001) had a highly significant impact on the prevalence of the parasite. In contrast, sampling sites (*P*= 0.096) had no significant influence, as expected from experimental design and Salathé and Schmid-Hempel (2011). Using model reduction by mean of stepwise backward removal, only host species and year remained inside the model, whereas sampling location was removed.

We selected a subset of biological markers (morphological data, foraging indices, etc.) for the respective bumblebee species from literature (von Hagen and Aichhorn 2003; Goulson and Darvill 2004) in order to evaluate an association of the parasite prevalence and species-specific ecological covariates.

**Figure 3:**
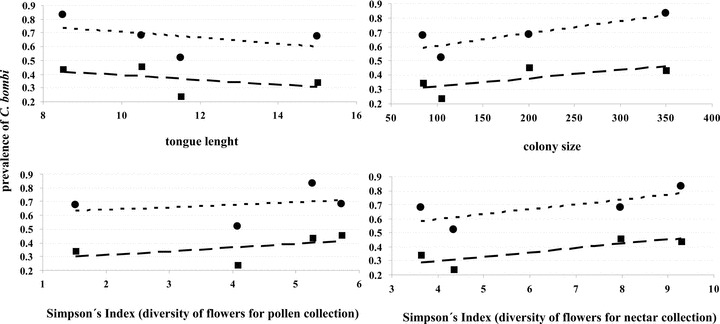
Correlation analysis of prevalence of *Crithidia bombi* and several ecological and life-history characters. Tongue length and colony sizes are adapted from von Hagen and Aichhorn (2003) and Simpson's indices were taken from Goulson and Darvill (2004). (Filled circles: samples 2009, filled squares: samples 2008; from left to right—tongue length: *Bombus terrestris*, *B. lapidarius*, *B. pascuorum*, and *B. hortorum*; colony size: *B. hortorum*, *B. pascuorum*, *B. lapidarius*, and *Bombus terrestris*; Simpson's index (pollen): *B. hortorum*, *B. pascuorum*, *Bombus terrestris*, and *B. lapidarius*; Simpson's index (nectar): *B. hortorum*, *B. pascuorum*, *B. lapidarius*, and *Bombus terrestris*; dashed lines: corresponding trend lines).

We found a negative association between tongue length and prevalence as well as positive association between prevalence and colony size and the diversity of flowers used for nectar collection (Simpson index of diversity, Goulson and Darvill 2004), respectively. The association of prevalence and flower diversity for pollen collection was less strong. However, none of these relationships appeared to be significant, most likely due to the restricted number of species available for analysis (Fig. 3).

## Discussion

Using the variation in the distribution of parasite genotypes, we tested the explanatory power of host genotype distribution, temporal variation, and spatial distribution to predict parasite population characteristics in a multi-host parasite system.

We found that the main factor shaping the population structure of the parasite is a temporal effect.

Host species did not show any significant effect on parasite genotype distribution. Geographic locations with different habitat structure as influencing factor may be another source for variation in parasite genotype distribution as shown recently by Salathé and Schmid-Hempel (2011).

There are three mutually non-exclusive explanations for this temporal effect: (1) the hibernation period of young queens represents a significant bottleneck for the *C. bombi* population, (2) the high mobility of males in late summer and autumn (Wolf et al. 2012) as well as the high mobility of young queens (Lepais et al. 2010) connects different populations and leads to a high migration rate of *C. bombi* genotypes, and (3) differences in the selection pressures acting on different *C. bombi* genotypes throughout the year (Popp et al. 2012).

The prevalence of *C. bombi* changes throughout the year. Hibernated, colony-founding queens revealed a prevalence level of approximately 10%, whereas the prevalence of *C. bombi* in workers and males steadily increases throughout the year (Imhoof and Schmid-Hempel 1999; Gillespie 2010; Popp et al. 2012).

Flight ranges of males and queens have been estimated to range from 2.6 to 9.9 km for males of *B. terrestris* (Kraus et al. 2009) and up to 3 or 5 km for queens of *B. lapidarius* and *B. pascuorum*, respectively (Lepais et al. 2010). These ranges typically exceed those of workers (267.2 ± 180.3 m, Wolf and Moritz 2008; reviewed in Goulson and Osborne 2009) and hence may contribute to wide range of dispersal of *C. bombi* genotypes.

Selection pressures on *C. bombi* genotypes may change throughout the year. Both, environmental conditions and transmission opportunities fundamentally change through the season from metabolically stressed hibernating or nest-founding queens, which are effectively solitary insects, to the homeostatic conditions of a large bumblebee colony with a large number of suitable hosts (workers) available. Additionally, selection pressure on *C. bombi* may be particularly strong in multiply infected hosts, either due to strain filtering (Ulrich et al. 2011) or due to competition between parasites strains (Popp et al. 2012).

Horizontal transmission on flowers (Durrer and Schmid-Hempel 1994) might increase throughout the year, as colonies grow under resource-rich conditions during spring and early summer, when contact rate on flowers increases due to an increase in abundance of foraging workers. *Crithidia bombi* transmission is further enhanced in bumblebee species sharing similar plants for foraging. Pollen- and nectar-foragers with short probosces (*B. terrestris*, *B. lapidarius*) share a broad spectrum of available flowers compared to long-probosces bumblebee species (*B. hortorum*, *B. pascuorum*) (Goulson and Darvill 2004). Higher prevalence of *C. bombi* might be explained by increased access to *C. bombi* contaminated flowers as well as with high intracolonial transmission in large colonies.

However, we found no evidence for adaptation to different host species when studying single *Crithidia*-infected bumblebees. Global analyses of all detected *C. bombi* genotypes and their hosts indicated slight association of parasite genotypes with their host species (Fig. 1). One switch on the side of the *C. bombi* populations occurred involving the *C. bombi* genotypes extracted from *B.* (*Psithyrus*) *vestalis*, the cuckoo bumblebee parasitizing *B. terrestris* (Loken 1984). This might be explained by the more frequent contact between these species due to the shared nest, increasing the interspecific transmission of *C. bombi* genotypes.

Very strong support for a host-parasite association was detected for specific multiple infections found in *B. lapidarius* and in *B. pascuorum*, respectively, where combinations (two) of the same *C. bombi* genotypes have been detected. Similar patterns for multiple infections across different species are rather common and are fixed by ecological factors (Salathé and Schmid-Hempel 2011). An alternative explanation might be, that these are triploid clones, derived during genetic exchange, as this phenomenon is known from *Leishmania major* (Akopyants et al. 2009). Unfortunately, it was not possible to verify this from a marker-based analysis.

In midsummer, the degree of multiple infections increases along with the overall prevalence (Popp et al. 2012), potentially increasing competition between different *C. bombi* genotypes. Under these conditions, sexual recombination might be beneficial, yet counteracting local adaptation. Asexual reproduction is assumed to be the main mode of propagation for the genus *Crithidia* (McGhee and Cosgrove 1980), but signs of sexual reproduction in putatively asexual trypanosomatid species are known from *L. major*, *Trypanosoma brucei*, and *T. cruzi* (Akopyants et al. 2009; Schurko et al. 2009). Recently, laboratory tests have shown that genetic exchange also occurs in *C. bombi* (Schmid-Hempel et al. 2011). Our data indicate that sexual reproduction as an alternative reproductive strategy also occurs under natural conditions, as suggested by Salathé and Schmid-Hempel (2011) and Schmid-Hempel and Reber Funk (2004). The high diversity of genotypes present in the population (total number of circulating genotypes: 119–242), lack of deviation from HWE, and no signs of linkage disequilibrium strongly suggest recurrent genetic exchange between strains.

This is even more so, considering the lifecycle of the host. *Crithidia bombi* is mainly transmitted from year to year via hibernating queens. As hibernating queens typically show low levels of prevalence (10–30%, Shykoff and Schmid-Hempel 1991b) and are predominantly singly infected (Ulrich et al. 2011), hibernation represents a strong bottleneck for the *C. bombi* population. This is strongly supported by only 33.3% of the detected genotypes being found in both years. Under these conditions, a strictly clonal reproduction is expected to lead to genetically deprived populations consisting of few genotypes only, which is in contrast to our findings. The analysis of the truly clonal *C. bombi* types, that is, types that have been detected more than once, revealed at least two different host species for the majority of *C. bombi* genotypes (78%) indicating high levels of interspecific transmission counteracting local adaptation toward host species.

Most of the previous studies that focused on local adaptation between *C. bombi* and its host usually paid attention to a single host species, *B. terrestris*. The results that have been found so far did not allow for a full picture of host-parasite interaction. However, by analyzing a broad host spectrum, we were able to show that local adaptation is very unlikely to occur within certain host species. Frequent horizontal transmission may be the main driver preventing local adaptation with respect to the two proposed mechanisms (genetic and temporal). In this study, we show that remarkable differences within the parasite population exist between years, but no genetic adaptation (distinct host species) was detectable.

## Supporting Information - Evolutionary Analysis

**Table S1.** Overview on diversity of bumblebee samples and amount of individual bumblebees and sex of bumblebees.

**Table S2.** Population genetic analysis of microsatelliteloci for *Bombus terrestris* (*B. t.*), *B.lapidarius* (*B. l.*), *B. hortorum* (*B. h.*),*B. pascuorum* (*B. p.*), and *B.*(*Psithyrus*) *vestalis* (*B. v.*) from both sitesand years and comparison of diversity indices between allspecies.

**Table S3.** Estimated pairwise *F*_ST_ values to characterize population differences between host and parasite populations.
