# Supplementary material for: Sex, horizontal transmission, and multiple hosts prevent local adaptation of Crithidia bombi, a parasite of bumblebees (Bombus spp.)
Source: Ecol Evol. 2012 May;2(5):930–40. doi: 10.1002/ece3.250 (PMC3399159; doi:10.1002/ece3.250)
Supplement: Supplementary file 1 [file ece30002-0930-SD1.pdf]

Supporting information:

Table S1: Overview on diversity of bumblebee samples and amount of individual bumblebees and sex of bumblebees. Reconstructed observed and expected colonies, both years and sites of sampling were included (BG: botanical garden, HS: Heide-Süd).

species	site	*B. terrestris*	*B. lapidarius*	*B. hortorum*	*B. pascuorum*	*B. vestalis*	*B. terrestris*	*B. lapidarius*	*B. hortorum*	*B. pascuorum*	*B. vestalis*
	BG	48	-	26	29	23	35	37	28	40	36
**sample size total**	HS	48	48	-	-	-	52	48	-	10	-
	BG	24 / 24	-	6 / 20	25 / 4	- / 23	22 / 13	13 / 24	4 / 24	24 / 16	0 / 36
**sample size sex (f / m)**	HS	24 / 24	24 / 24	-	-	-	36 / 16	24 / 24	-	10 / -	-
	BG	24	-	13	11	12	20	14	13	12	11
**colonies observed**	HS	22	23	-	-	-	23	17	-	4	-
	BG	27.2	-	14.3	11.9	13.8	24.2	15.5	15.4	12.6	11.5
**colonies expected**	HS	24.5	25.9	-	-	-	27.4	18,7	-	4.4	-
**sampling year**		2008	2008	2008	2008	2008	2009	2009	2009	2009	2009

f: females (worker); m: males (drones)

Table S2: Population genetic analysis of microsatellite loci for *Bombus terrestris (B. t.), B. lapidarius (B. l.), B. hortorum (B. h.), B. pascuorum (B. p.)* and *B. (Psithyrus) vestalis (B. v.)* from both sites and years and comparison of diversity indices between all species.

**locus**				**B10**	**B100**	**B11**	**B124**	**B126**
**allelic richness**	**2008**	*B. t.*	BG	5.33	4.01	4.07	5.00	4.60
		B. t.	HS	5.58	4.43	4.55	5.25	5.26
		B. l.	HS	5.41	4.36	5.52	4.18	4.96
		B. h.	BG	3.89	4.01	3.60	3.67	4.55
		B. p.	BG	2.59	1.36	2.70	4.72	2.82
		B. v.	BG	4.06	4.32	1.00	1.00	3.62
	**2009**	*B. t.*	BG	5.51	3.85	4.00	5.49	4.93
		B. t.	HS	4.63	3.90	4.13	4.96	5.10
		B. l.	BG	4.13	5.23	4.76	5.05	4.42
		B. l.	HS	4.73	5.04	4.84	3.85	4.70
		B. h.	BG	4.77	4.85	4.36	4.39	4.68
		B. p.	BG	1.67	1.00	2.70	4.66	3.12
		B. p.	HS	2.00	1.00	1.00	5.00	4.00
		B. v.	BG	4.21	5.62	1.76	1.00	2.98
**No. of alleles and (n)**	**2008**	*B. t.*	BG	20 (72)	12 (72)	10 (72)	16 (72)	14 (72)
		B. t.	HS	18 (67)	12 (68)	12 (72)	14 (72)	15 (71)
		B. l.	HS	11 (62)	10 (72)	16 (72)	12 (72)	11 (68)
		B. h.	BG	8 (27)	8 (30)	8 (30)	7 (32)	11 (30)
		B. p.	BG	5 (49)	2 (46)	8 (51)	6 (53)	5 (17)
		B. v.	BG	8 (21)	7 (18)	2 (23)	1 (23)	6 (23)
	**2009**	*B. t.*	BG	20 (43)	12 (49)	13 (49)	13 (49)	13 (51)
		B. t.	HS	21 (52)	10 (63)	12 (65)	16 (63)	17 (65)
		B. l.	BG	10 (36)	9 (43)	15 (44)	11 (44)	9 (44)
		B. l.	HS	13 (43)	9 (60)	13 (59)	7 (57)	9 (60)
		B. h.	BG	9 (38)	10 (40)	9 (40)	10 (39)	12 (39)
		B. p.	BG	5 (44)	2 (45)	6 (43)	7 (44)	4 (45)
		B. p.	HS	3 (18)	1 (18)	3 (18)	6 (18)	5 (16)
		B. v.	BG	6 (28)	9 (28)	2 (33)	2 (31)	7 (31)
H_E	**2008**	*B. t.*	BG	0.85	0.71	0.75	0.83	0.79
		B. t.	HS	0.88	0.76	0.78	0.86	0.87
		B. l.	HS	0.87	0.77	0.86	0.73	0.84
		B. h.	BG	0.71	0.72	0.58	0.62	0.76
		B. p.	BG	0.45	0.09	0.41	0.84	0.55
		B. v.	BG	0.69	0.76	0	0	0.61
	**2009**	*B. t.*	BG	0.86	0.67	0.71	0.87	0.82
		B. t.	HS	0.74	0.69	0.74	0.82	0.83
		B. l.	BG	0.67	0.87	0.77	0.85	0.81
		B. l.	HS	0.81	0.86	0.79	0.71	0.83
		B. h.	BG	0.80	0.80	0.71	0.74	0.75
		B. p.	BG	0.16	0	0.43	0.82	0.58
		B. p.	HS	0.25	0	0	0.79	0.75
		B. v.	BG	0.78	0.89	0.25	0	0.52

***H**$_O$*	**2008**	*B. t.* BG	0.79	0.71	0.67	0.79	0.83
		B. t. HS	0.77	0.86	0.86	1	0.73
		B. l. HS	0.91	0.78	0.91	0.70	0.96
		B. h. BG	0.58	0.69	0.62	0.62	0.77
		B. p. BG	0.55	0.09	0.36	0.64	0.33
		B. v. BG	0.75	0.73	0	0	0.58
	**2009**	*B. t.* BG	0.88	0.74	0.79	0.79	0.80
		B. t. HS	0.81	0.70	0.74	0.91	0.91
		B. l. BG	0.71	0.79	0.71	0.71	0.50
		B. l. HS	0.69	0.76	0.71	0.82	0.88
		B. h. BG	0.69	0.77	0.83	0.69	0.77
		B. p. BG	0.17	0	0.50	0.75	0.58
		B. p. HS	0.25	0	0	0.75	0.50
		B. v. BG	0.64	0.82	0.27	0	0.45
**size range (bp)**	**2008**	*B. t.* BG	175-221	149-171	152-174	239-271	169-211
		B. t. HS	187-217	145-171	154-178	243-273	196-201
		B. l. HS	203-227	151-173	142-190	233-277	131-163
		B. h. BG	173-185	145-163	136-168	243-277	175-205
		B. p. BG	173-191	137-141	128-162	231-259	145-173
		B. v. BG	193-215	137-171	140	233-253	153-169
	**2009**	*B. t.* BG	167-217	137-177	134-174	233-269	135-201
		B. t. HS	181-247	149-169	140-174	233-281	145-193
		B. l. BG	205-231	149-169	144-188	243-277	143-157
		B. l. HS	203-239	153-173	144-190	265-285	139-163
		B. h. BG	173-209	145-171	122-164	233-277	153-201
		B. p. BG	171-193	137-143	130-140	249-279	125-131
		B. p. HS	173-179	137	130-138	249-261	125-135
		B. v. BG	193-209	145-181	140-142	233-249	151-167

(n): number of individuals tested per loci; H_E: expected heterozygosity; H_O: observed heterozygosity; BG: botanical garden; HS: Heide-Süd

Table S3: Estimated pairwise F_{ST} values to characterise population differences between host and parasite populations. The upper triangular illustrates F_{ST} values for the *C. bombi* populations and the lower for the corresponding bumblebee populations. Negative F_{ST} values probably reflect minor inaccuracies of the algorithm used by the software to estimate these values.

Parasite / Host	***B. t.* HS 08**	***B. t.* BG 08**	***B. t.* BG 09**	***B. t.* HS 09**	***B. l.* HS 08**	***B. l.* BG 09**	***B. l.* HS 09**	***B. h.* BG 09**	***B. h.* BG 08**	***B. p.* BG 08**	***B. p.* BG 09**	***B. p.* HS 09**	***B. v.* BG 08**	***B. v.* BG 09**
***B. t.* HS 08**		-0.006	0.036	0.015	0.002	0.012	0.035	0.012	-0.018	-0.007	0.053	-0.051	0.049	-0.018
***B. t.* BG 08**	0.121		0.049	0.013	-0.025	0.018	0.048	0.048	0.027	-0.030	0.140	-0.038	0.059	0.030
***B. t.* BG 09**	0.027	0.121		-0.046	0.021	0.008	0.004	-0.023	0.098	-0.043	-0.042	-0.040	0.074	-0.015
***B. t.* HS 09**	0.084	0.111	0.069		-0.023	-0.008	-0.037	0.030	0.094	-0.123	-0.073	-0.112	0.074	-0.004
***B. l.* HS 08**	0.172	0.166	0.177	0.187		-0.018	0.010	0.009	0.049	-0.022	0.045	-0.109	0.061	0.019
***B. l.* BG 09**	0.185	0.179	0.190	0.191	0.085		0.006	-0.002	0.029	-0.015	0.015	-0.009	0.054	-0.006
***B. l.* HS 09**	0.183	0.179	0.185	0.190	0.081	0.009		0.012	0.056	-0.037	-0.020	-0.028	0.075	0.033
***B. h.* BG 09**	0.207	0.187	0.209	0.223	0.165	0.171	0.148		0.067	-0.020	0.012	0.010	0.060	0.000
***B. h.* BG 08**	0.257	0.240	0.263	0.270	0.207	0.230	0.220	0.225		-0.001	0.189	0.117	0.070	0.033
***B. p.* BG 08**	0.340	0.318	0.348	0.354	0.330	0.355	0.353	0.366	0.424		-0.098	-0.286	-0.005	0.003
***B. p.* BG 09**	0.369	0.349	0.376	0.383	0.365	0.391	0.386	0.401	0.458	0.305		0.184	0.120	-0.020
***B. p.* HS 09**	0.351	0.322	0.351	0.366	0.340	0.356	0.356	0.365	0.434	0.319	0.018		-0.001	-0.035
***B. v.* BG 08**	0.376	0.321	0.385	0.385	0.359	0.389	0.383	0.404	0.456	0.569	0.590	0.607		0.044
***B. v.* BG 09**	0.316	0.320	0.320	0.319	0.315	0.341	0.333	0.351	0.413	0.523	0.550	0.553	0.283	

B. t.: *B. terrestris*; *B. l.*: *B. lapidarius*; *B. h.*: *B. hortorum*; *B. p.*: *B. pascuorum*; *B. v.*: *B. vestalis*; BG: botanical garden; HS: Heide-Süd; 08, 09: sampling years 2008 and 2009
